# Endoscopic and Conservative Management of Symptomatic Lemmel Syndrome: A Systematic Review and Patient-Level Synthesis

**DOI:** 10.1055/a-2979-6121

**Published:** 2026-10-28

**Authors:** Muhammad Arsalan, FNU Sawaira, Muhammad Mukhlis, Malaika Khan, Suliman Shah, Haris Mumtaz Malik, Zain Shahzad, Tarooba Khan, Muhammad Yasir, Gauhar Ullah, Muhammad Hassan Saeed, Abdul Muiz, Roshan Shrestha

**Affiliations:** 1 Department of Internal MedicineSt Luke’s HospitalChesterfieldUnited States; 2 Department of MedicineKhyber Girls Medical CollegePeshawarPakistan; 3 Department of MedicineAyub Medical CollegeAbbottabadPakistan; 4 Department of MedicineZiauddin Medical CollegeKarachiPakistan; 5 Department of MedicineQazi Hussain Ahmad Medical ComplexNowsheraPakistan; 6 Department of MedicineRawalpindi Medical UniversityRawalpindiPakistan; 7 Department of MedicineAllama Iqbal Medical CollegeLahorePakistan; 8 Department of MedicineJinnah Sindh Medical UniversityKarachiPakistan; 9 Department of MedicineBacha Khan Medical CollegeMardanPakistan; 10 Department of Medicine 66942 Khyber Medical CollegePeshawarPakistan; 11 Department of MedicineJinnah Postgraduate Medical CenterKarachiPakistan; 12 Department of GastroenterologySt Luke’s HospitalChesterfieldUnited States

**Keywords:** lemmel syndrome, periampullary diverticulum, ERCP, obstructive jaundice, systematic review, individual participant data

## Abstract

**Background** Lemmel syndrome is obstructive jaundice or pancreaticobiliary ductal dilatation caused by extrinsic compression from a periampullary duodenal diverticulum, without choledocholithiasis or periampullary neoplasm. Management is not standardised, and no patient-level synthesis of outcomes exists.

**Materials and Methods** We searched PubMed, Embase, and the Cochrane Library for adults with symptomatic Lemmel syndrome reporting management and outcome, synthesised at patient level with exact Clopper–Pearson confidence intervals. Co-primary outcomes were endoscopic retrograde cholangiopancreatography (ERCP) technical success and durable endoscopic success, defined as resolution without surgical or percutaneous rescue; recurrence-free survival was estimated using Kaplan–Meier analysis.

**Results** Twenty-six studies reporting 46 patients were identified; one diagnostic series and one non-qualifying patient were excluded, leaving 26 patients from 24 reports. ERCP was attempted in 16 patients (61.5%), with technical success in 14 (87.5%) and endoscopic success in 12 (75.0%); four (25.0%) required rescue. One bleeding event occurred (6.3%); no pancreatitis, perforation, or death was reported. Among 20 patients with follow-up, three recurrences occurred; Kaplan–Meier recurrence-free survival was 93.3% at 1 month, 85.6% at 1.84 months, and 73.3% at 6 months.

**Conclusion** Endoscopic therapy achieves technical success, although one in four require rescue. Kaplan–Meier analysis estimated 26.7% recurrence by 6 months; prospective registry data with longer follow-up are needed.

## Introduction

Lemmel syndrome is defined as obstructive jaundice or biliary and pancreatic ductal dilation caused by extrinsic compression of the distal common bile duct from a periampullary duodenal diverticulum (PAD), occurring in the absence of choledocholithiasis or a periampullary tumour. 1 2 The condition was first described by the German physician Gerhard Lemmel in 1934. 1 Its pathophysiology is not fully understood, but three overlapping mechanisms have been proposed: direct mechanical compression of the distal bile duct or ampulla by the diverticulum itself or by food debris and enteroliths retained within it; diverticulum-induced dysfunction of the sphincter of Oddi; and chronic inflammation of the ampulla progressing to fibrosis and stenosis. 2 3 4 Based on the position of the major duodenal papilla relative to the diverticulum, PAD is classified as type I (papilla within the diverticulum), type II (papilla at its margin), or type III (papilla near the diverticulum). 2

PAD is a common incidental finding, identified in approximately 5–33% of patients undergoing endoscopic retrograde cholangiopancreatography (ERCP) and in up to 23–32% of autopsy series. 5 6 Only about 1–5% of PAD cases ever become clinically symptomatic. 3 7 Presentation is nonspecific and may include jaundice, right-upper-quadrant or epigastric pain, cholangitis, or pancreatitis. Symptoms can be intermittent, as debris retained within the diverticulum transiently obstructs and then releases the ampulla. 8

Because its clinical and biochemical features overlap substantially with choledocholithiasis, cholangiocarcinoma, and pancreatic or ampullary malignancy, Lemmel syndrome is frequently misdiagnosed, leading to unnecessary cholecystectomy or resection performed for presumed malignancy. 2 9 Transabdominal ultrasonography often fails to identify the diverticulum, whereas contrast-enhanced computed tomography and magnetic resonance cholangiopancreatography better delineate the periampullary anatomy and help exclude neoplastic disease. ERCP remains the reference standard, as it allows direct visualisation of the diverticulum and papilla together with the option of immediate therapeutic intervention. 2 9

Management has not been standardised. Reported strategies range from conservative management and antimicrobial therapy for cholangitis to ERCP-based endoscopic therapy, which involves biliary sphincterotomy with or without balloon clearance of diverticular contents and temporary stent placement. Surgical options such as diverticulectomy or biliodigestive bypass are reserved for endoscopic failure or diverticulum-related complications. 2 9 ERCP-based therapy is widely regarded as the preferred first-line approach, given its combined diagnostic and therapeutic role and its lower morbidity relative to surgery. 2 9 However, ERCP is not risk-free: pooled data indicate an incidence of post-ERCP pancreatitis of approximately 4.6%, cholangitis 2.5%, bleeding 1.5%, and perforation 0.5%. 10 The presence of a periampullary diverticulum can itself complicate papillary identification and cannulation. 5 Whether these figures hold for the older, often cholestatic or septic population that presents with Lemmel syndrome has not been formally established.

The largest existing synthesis of published cases is a narrative literature review appended to a case report, containing 46 cases. 2 That review reported treatment approach and short-term outcome descriptively; it did not define technical or durable success, did not analyse recurrence as a time-to-event outcome, and did not report denominators for adverse events. Because it aggregates cases from primary reports, using it as a data source risks double counting; we therefore extracted only its index case.

We conducted this systematic review to characterise the management and outcomes of adults with symptomatic Lemmel syndrome. Our specific objectives were to establish how frequently ERCP is actually delivered in reported cases; to estimate technical success, durable endoscopic success and the need for surgical or percutaneous rescue among those who receive it; to estimate recurrence accounting for censoring; to quantify procedure-related harm with appropriate denominators; and to document the completeness of outcome reporting in the existing literature.

## Materials and Methods

### Protocol and Registration

This systematic review followed the PRISMA 2020 statement 11 and the Cochrane Handbook for Systematic Reviews of Interventions. 12 The protocol specified a meta-analysis of proportions; this was changed to a patient-level synthesis after data extraction, when it became apparent that almost every source contributed a single patient. Assessment of certainty of evidence using GRADE was added post hoc. Both deviations are reported here in accordance with PRISMA item 24c.

### Search Strategy

We searched PubMed, Embase, and the Cochrane Library from inception to July 2026. Search terms combined variants of (“Lemmel syndrome” OR “periampullary diverticulum” OR “juxtapapillary diverticulum”) AND (“biliary obstruction” OR “cholangitis” OR “jaundice”) AND (“ERCP” OR “endoscopic retrograde cholangiopancreatography”). No language restriction was applied and no report was excluded on language grounds. Reference lists of included articles were hand-searched. Duplicate records were removed before title-and-abstract screening; titles and abstracts, and then full texts, were screened independently and in duplicate against the eligibility criteria, with disagreements resolved by discussion and consensus.

### Eligibility Criteria and Case Adjudication

#### Inclusion Criteria

- Adults with symptomatic Lemmel syndrome confirmed radiologically and/or endoscopically.
- Management reported with an assessable clinical outcome.

#### Exclusion Criteria

- Lemmel syndrome patients who could not be separated from a mixed periampullary-diverticulum population.
- Insufficient outcome data.
- Intervention not described.

#### Case Definition (All Three Required)

- A periampullary or juxtapapillary duodenal diverticulum demonstrated on imaging or endoscopy.
- Biliary obstruction evidenced by jaundice, cholangitis, or ductal dilatation.
- Documented exclusion of choledocholithiasis and periampullary neoplasm.

Patients failing any criterion were excluded and the reason recorded. Sources reporting cases aggregated from other publications contributed only their own index case, and where the same patient appeared in more than one report the earliest primary report was retained.

### Data Extraction and Outcome Definitions

Data were extracted independently and in duplicate using a piloted, standardised form covering study identification and design, patient demographics and presentation, diagnostic imaging, intervention details, outcomes and follow-up; discrepancies were reconciled against the source article. Missing data were coded as not reported (NR) rather than left blank. Two co-primary outcomes were defined. Technical success was successful biliary cannulation with completion of the intended endoscopic intervention at the index ERCP. Durable endoscopic success was resolution of jaundice or cholangitis during the index admission without unplanned surgical or percutaneous rescue and without repeat intervention for the same episode. Patients requiring rescue were classified as failures of the index endoscopic intervention irrespective of their eventual clinical outcome. Secondary outcomes were recurrence, repeat intervention, post-ERCP pancreatitis, bleeding, perforation, any procedure-related complication, and 30-day mortality. Biochemical outcomes were assessed only in patients with an abnormal baseline value, since normalisation is undefined where the baseline is normal.

### Statistical Analysis

Because all but two included publications contributed a single patient, a two-stage meta-analysis of proportions was not appropriate: with one participant per study, each study contributes a point estimate of 0 or 1 with no within-study variance, and the inverse Freeman–Tukey double-arcsine back-transformation is known to give seriously misleading results under these conditions. 13 We therefore conducted a one-stage individual-participant-data synthesis. Outcomes are reported as patient-level proportions with exact (Clopper–Pearson) 95% confidence intervals. 14 Because the outcomes were binary, denominators were small and several outcomes had few or zero events, exact Clopper–Pearson intervals were used. This method is conservative and tends to produce somewhat wider intervals than alternative exact procedures such as the Blyth–Still–Casella interval, 15 16 which can provide narrower intervals while maintaining nominal coverage. Given the sparse nature of the available data, we considered it more appropriate to report conservative interval estimates than to risk overstating precision.

Denominators were defined for each outcome and restricted to patients in whom the outcome was both defined and reported; procedure-related outcomes are denominated on ERCP recipients rather than the whole cohort. The absence of any mention of an adverse event was coded as NR, not as the absence of the event. Recurrence was analysed by the Kaplan–Meier method 17 with censoring at last reported contact and confidence intervals from Greenwood’s formula on the complementary log–log scale; this analysis was performed on the whole cohort. For outcomes with zero events, the exact upper confidence bound is reported and interpreted as the highest event rate compatible with the data. Analyses were performed in Python 3 (SciPy 1.17). No pooled meta-analytic estimates and no heterogeneity statistics are reported, as neither is interpretable in this data structure.

The Kaplan–Meier method assumes non-informative censoring, an assumption that may not be fully met in a cohort assembled from case reports and case series. Patients with uncomplicated clinical courses may have had limited follow-up, whereas patients experiencing recurrence may have undergone longer observation, introducing the potential for informative censoring. Consequently, recurrence-free survival estimates may underestimate the true recurrence risk and should be interpreted as descriptive of the available follow-up rather than definitive estimates of long-term recurrence.

### Certainty of Evidence

Certainty of evidence was rated for each outcome using GRADE 18 and is summarised in a Summary of Findings table. As non-comparative observational data, all outcomes began at low certainty and were considered for downgrading for risk of bias, imprecision, publication bias, indirectness, and inconsistency; recurrence was additionally downgraded for potential informative censoring. Rating up was not appropriate without a comparator, and favourable Joanna Briggs Institute (JBI) reporting scores were not used to raise certainty.

### Risk of Bias Assessment

Methodological quality was appraised independently and in duplicate (disagreements resolved by consensus) using the JBI Critical Appraisal Checklist for Case Reports (eight items) for individual cases and the JBI Checklist for Case Series (ten items) for the single case series. 19 Items were scored Yes, Unclear, or No, with silence on adverse events scored Unclear; an overall low, moderate or high judgement was assigned and displayed as traffic-light and summary plots. As these tools gauge reporting completeness rather than internal validity, the appraisal informed the GRADE risk-of-bias domain rather than any weighting or exclusion of data, and a sensitivity analysis excluding moderate- or higher-risk reports was pre-specified for the co-primary outcomes.

## Results

### Study Selection

The search identified 813 records from PubMed ( *n* = 114), Embase ( *n* = 668), and the Cochrane Library ( *n* = 31). After removal of 412 duplicate records, 401 were screened by title and abstract and 364 were excluded. Thirty-seven full-text reports were sought, all were retrieved, and all were assessed for eligibility. Eleven were excluded: duplicate publication of a case already captured by an included report ( *n* = 5), irrelevant patient population ( *n* = 1), insufficient outcome data ( *n* = 2), and no description of the ERCP-based intervention ( *n* = 3). Twenty-six studies met the inclusion criteria ( Fig. 1 ).

**Fig. 1:**
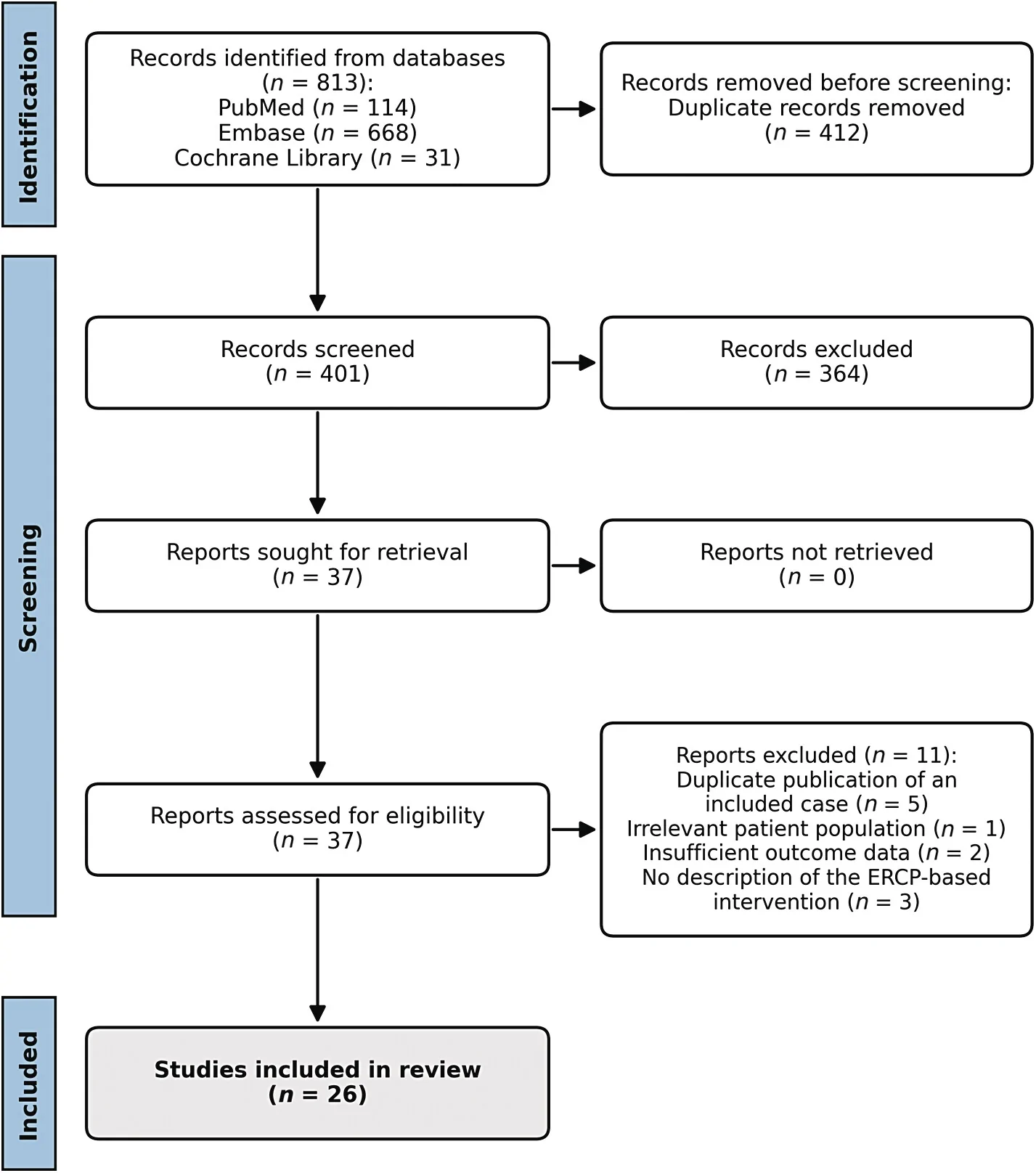
PRISMA 2020 flow diagram of study identification, screening, and inclusion, prepared in accordance with the PRISMA 2020 statement. Source: Reproduced from Page MJ et al. 11 Licensed under CC BY 4.0.

### Analysis Cohort

The 26 included publications reported 46 patients. Two sources were excluded from treatment-outcome analyses. Chen 2025 20 ( *n* = 19) is a retrospective diagnostic endoscopic-ultrasonography study that reports no treatment category, no therapeutic outcome, and no follow-up; its patients are not included in any denominator. One further patient did not meet the case definition, having metastatic colorectal carcinoma with a post-operative biliary leak and subhepatic abscess rather than diverticulum-related obstruction. The analysis cohort therefore comprises 26 patients from 24 publications ( Table 1 ).

**Table 1:** Characteristics of the 26 studies included in the review, with the two excluded from the analysis cohort indicated.

ID	First author	Year	Journal	Country	Design	N	In analysis	Ref
S01	Chen	2025	PLoS ONE	China	Retrospective case series	19	No—excluded	20
S02	Garcia Garza	2025	Cureus	Mexico	Case report	1	Yes	21
S03	Hanaoka	2025	Cureus	Japan	Case report	1	Yes	22
S04	Hadadia	2025	Cureus	Morocco	Case report	1	Yes	23
S05	Wyke	2026	IJMSCRS	Myanmar	Case report	1	Yes	24
S06	Subramonian	2024	Cureus	India	Case report	1	Yes	25
S07	Love	2022	Case Rep Gastroenterol	USA	Case report + pooled review	1	Yes	2
S08	Alzerwi	2020	Medicine (Baltimore)	Saudi Arabia	Case report	1	Yes	26
S09	Azzam	2021	Cureus	Saudi Arabia	Case report	1	Yes	27
S10	Lopez	2023	Cureus	Mexico	Case series	2	Yes	28
S11	Suveran	2025	Cureus	Turkey	Case report	1	No—excluded	29
S12	Anebo	2026	Clin Case Rep	Spain	Case report	1	Yes	30
S13	Khan	2017	EJCRIM	UK	Case report	1	Yes	31
S14	Bellamlih	2021	BJR Case Rep	Morocco	Case report	1	Yes	32
S15	Bernshteyn	2020	Cureus	USA	Case report	1	Yes	33
S16	Yanagaki	2020	Int J Surg Case Rep	Japan	Case report	1	Yes	34
S17	Tabata	2020	Pol Arch Intern Med	Japan	Clinical image	1	Yes	35
S18	Frauenfelder	2019	J Clin Imaging Sci	Italy	Case series	2	Yes	36
S19	Venkatanarasimha	2019	Radiology	Singapore	Educational case (Diagnosis Please)	1	Yes	37
S20	Oliveira	2019	BMJ Case Rep	Portugal	Case report	1	Yes	38
S21	Miyajima	2018	IDCases	Japan	Case report	1	Yes	39
S22	Somani	2017	Indian J Gastroenterol	India	Case report	1	Yes	40
S23	Rouet	2012	Clin Res Hepatol Gastroenterol	France	Case report	1	Yes	41
S24	Kang	2014	J Korean Med Sci	South Korea	Case report	1	Yes	42
S25	Tobin	2018	J Surg Case Rep	Ireland	Case report	1	Yes	43
S26	Desai	2017	Cureus	USA	Case report	1	Yes	4

### Study and Patient Characteristics

The included reports originated from 17 countries across four continents and were published between 2012 and 2026, with 20 of 26 appearing since 2019, consistent with increasing recognition of the condition. Twenty-two were single case reports, three were case series of two or nineteen patients, and one was a case report accompanied by a pooled literature review. Each included study is cited individually 21 22 23 24 25 26 27 28 29 30 31 32 33 34 35 36 37 38 39 40 41 42 43 and cross-referenced to its identifier in Table 1 .

Publication years were verified against the source articles rather than against the extraction record. Two required correction: Kang is published in J Korean Med Sci 2014;29(6):874–8 and Tobin in J Surg Case Rep 2018;2018(10):rjy263, and both are reported here with those years. Two reports are also not conventional case reports: Tabata is published as a Clinical Image and Venkatanarasimha as an educational “Diagnosis Please” case. Both remain eligible, since each describes an identifiable patient with management and outcome, but the JBI case-report checklist fits them imperfectly and their quality scores should be read with that in mind.

Baseline characteristics of the analysis cohort are shown in Table 2 . Median age was 73 years (range 24–94) and 14 of 26 patients (53.8%) were female. Jaundice was the commonest presenting feature ( *n* = 19), followed by abdominal pain ( *n* = 18), cholangitis ( *n* = 13), and pancreatitis ( *n* = 4); two patients had no symptom recorded in any category and are flagged in Table 2 , as they arguably do not meet the “symptomatic” criterion. Reported comorbidities included hypertension, diabetes mellitus, cholelithiasis, hepatic steatosis, and previous biliary intervention. The Boix classification was reported in only two patients and diverticulum size in 15; these variables were too incompletely reported to relate to outcome.

**Table 2:** Baseline characteristics of the 26 patients in the analysis cohort.

ID	Patient	Age	Sex	Presentation	Comorbidities	Diverticulum	Size (mm)	CBD (mm)	Bilirubin at presentation
S02	Garcia Garza 2025	62	M	Jaundice, cholangitis, pain	Grade III hepatic steatosis; recent cholecystitis/cholecystectomy	Periampullary diverticulum	NR	Ultrasound 10 mm, MRCP 14 mm, ERCP 12 mm	TB 9.8 mg/dL, DB 8.3 mg/dL, IB 1.5 mg/dL
S03	Hanaoka 2025	67	M	Jaundice, pain	Hypertension; heavy alcohol consumption	Periampullary diverticulum	NR	NR	Total bilirubin 5 mg/dL; Direct bilirubin 3.2 mg/dL
S04	Hadadia 2025	72	M	Jaundice, pain	NR	Periampullary diverticulum	NR	NR	Total bilirubin 26.8 mg/dL; Direct bilirubin 21.5 mg/dL
S05	Wyke 2026	74	M	Jaundice, cholangitis, pain	Grade I diastolic dysfunction; mild left ventricular hypertrophy; mild esophagitis and gastritis	Periampullary diverticulum	~30 × 20 mm (3 × 2 cm, intraoperative measurement)	16 mm	NR
S06	Subramonian 2024	~50s	F	Pain	Cholelithiasis (large gallstone); previous left cataract surgery; abnormal lipid profile	Periampullary	~11 mm	13 mm	NR
S07	Love 2022	82	F	Pain	Breast cancer (treated), dementia, diabetes mellitus, hypertension	Periampullary diverticulum	2.7 × 3.7 × 5.2 cm	10 mm	TB 0.6 mg/dL, DB 0.1 mg/dL
S08	Alzerwi 2020	24	F	Jaundice, cholangitis, pancreatitis	Anaemia (microcytic hypochromic, Hb 9.1 g/dL); hypoalbuminaemia; pre-renal azotaemia; no prior abdominal surgery	Periampullary	~50 mm	23 mm	Total bilirubin 114 μmol/L; Direct bilirubin 98 μmol/L (μmol/L)
S09	Azzam 2021	62	M	Pain	Gallstones without cholecystitis; moderate hepatic steatosis	Periampullary	~5 mm	16 mm	NR
S10	Lopez 2023 Case 1	94	F	Jaundice, cholangitis, pancreatitis, pain	Hypertension, prior open cholecystectomy	Juxtapapillary duodenal diverticulum	NR	11 mm	TB 7.68 mg/dL, DB 5.12 mg/dL
S10	Lopez 2023 Case 2	71	F	Jaundice, cholangitis, pain	Diabetes mellitus; hypothyroidism; prior cholecystectomy; prior multiple ERCPs with stent placements	Periampullary (juxtapapillary)	NR	16.3 mm	TB 1.2 mg/dL; DB 0.9 mg/dL
S12	Anebo 2026	90	F	Pain	Hypertension; hyperlipidaemia; osteoporosis; GERD; diverticulosis	Periampullary	25 mm	17 mm proximally, tapering to 7 mm distally	T bili 25.7 μmol/L (direct 10.3 μmol/L)
S13	Khan 2017	69	M	Jaundice, pancreatitis, pain	NR	Periampullary	~11 mm	NR	Total bilirubin 2.9 mg/dL; Direct bilirubin 1.4 mg/dL
S14	Bellamlih 2021	77	F	Jaundice, pain	Cholecystectomy (prior); hypertension; osteoarthritis	Periampullary (medial wall second portion duodenum)	23 × 18 mm (primary); 25 × 21 mm (second diverticulum)	12 mm	TB 6 mg/dL; DB 3.7 mg/dL
S15	Bernshteyn 2020	57	M	Jaundice, cholangitis, pain	Type II DM; GERD; psoriasis; prior laparoscopic cholecystectomy complicated by liver abscess and *Escherichia coli* bacteraemia	Periampullary (second portion duodenum)	20 mm	12 mm	TB 5.5 mg/dL
S16	Yanagaki 2020	81	F	Jaundice, cholangitis, pancreatitis, pain	No significant prior medical history	Periampullary (parapapillary diverticulum)	NR	Dilated CBD	TB 1.0 mg/dL
S17	Tabata 2020	91	F	Jaundice, cholangitis	Hypertension; ischaemic heart disease	Juxtapapillary (large juxtapapillary diverticulum)	36 mm	10 mm	TB 3.7 mg/dL
S18	Frauenfelder 2019 Case 1	79	M	Pain	NR	Periampullary (medial wall D2/D3 junction)	27 mm	11 mm	NR
S18	Frauenfelder 2019 Case 2	70	F	Jaundice, pain	NR	Periampullary (medial wall D2/D3 junction)	17 mm	13 mm	NR
S19	Venkatanarasimha 2019	70	M	Jaundice, cholangitis, pain	Gastric carcinoma (Billroth II + cholecystectomy 17 years prior); recurrent ascending cholangitis 6–12 months prior	Periampullary (medial aspect second/third portion duodenum)	NR	NR	TB 3.5 mg/dL (59.9 μmol/L)
S20	Oliveira 2019	89	M	Jaundice, cholangitis, pain	Dementia; osteoarthritis; medications: analgesics, donepezil, benzodiazepine	Juxtapapillary (medial wall second portion duodenum)	22 × 17 mm on CT; 25 mm on MRCP	7 mm	TB 5.1 mg/dL; DB 2.9 mg/dL
S21	Miyajima 2018	80	F	Jaundice, cholangitis	Hepatocellular carcinoma (prior partial hepatectomy); gallbladder cancer (prior cholecystectomy); diabetes mellitus; mild interstitial pulmonary fibrosis	Juxtapapillary (large juxtapapillary duodenal diverticulum)	NR	NR	TB 6.2 mg/dL
S22	Somani 2017	78	M	Jaundice	NR	Juxtapapillary/periampullary	NR	10 mm	TB 5.5 mg/dL
S23	Rouet 2012	70	M	Jaundice, cholangitis	Cholecystectomy 17 years earlier	Juxtapapillary (periampullary)	20 mm	NR	154 μmol/L
S24	Kang 2014	81	F	Jaundice, cholangitis	Subtotal gastrectomy with Billroth II for peptic ulcer perforation	Periampullary diverticulum	NR	NR	2.37 mg/dL
S25	Tobin 2018	80	F	none recorded	Known periampullary giant duodenal diverticulum (pre-existing); nursing home resident; end colostomy; para-stomal hernia; lithium-induced renal microcystic changes	Periampullary (D2—giant diverticulum)	18 mm	NR	TB 27.4 μmol/L on admission (units as reported); ALT 741; ALP 517; GGT 426
S26	Desai 2017	25	F	none recorded	No significant past medical history	Periampullary (medial wall second portion duodenum)	NR	18 mm	NR

### Delivery of the Index Intervention

ERCP was attempted in 16 of 26 patients (61.5%). The remaining ten were managed conservatively ( *n* = 7), conservatively followed by surgery ( *n* = 1), with antibiotics and percutaneous transhepatic drainage ( *n* = 1), or with non-ERCP endoscopic lavage at upper gastrointestinal endoscopy ( *n* = 1). Efficacy and safety estimates for ERCP are therefore based on 16 procedures, and this denominator is used throughout ( Table 3 ).

**Table 3:** Intervention and outcomes for the 26 patients in the analysis cohort.

ID	Patient	Treatment category	ERCP	Tech. success	Technique	Rescue	Recurrence	Follow-up	Adverse events
S02	Garcia Garza 2025	Endoscopic	Yes	Yes	ES, plastic stent, precut	No	No	0.2 mo	Bleeding (precut, procedure abandoned)
S03	Hanaoka 2025	Endoscopic	Yes	Yes	ES, balloon	No	NR	0.2 mo	None reported
S04	Hadadia 2025	Endoscopic	Yes	Yes	ES, plastic stent	No	No	6.0 mo	None reported
S05	Wyke 2026	Endoscopic failure → surgery	Yes	No	—	Yes	NR	NR	None reported
S06	Subramonian 2024	Endoscopic	Yes	Yes	ES, plastic stent	No	No	1.4 mo	Not reported
S07	Love 2022	Non-ERCP endoscopic (EGD lavage)	No	NA	—	No	No	1.0 mo	Not reported
S08	Alzerwi 2020	Endoscopic failure → surgery	Yes	No	—	Yes	No	24.0 mo	None reported
S09	Azzam 2021	Endoscopic	Yes	Yes	ES, plastic stent	No	No	6.0 mo	None reported
S10	Lopez 2023 Case 1	Endoscopic	Yes	Yes	ES, balloon, plastic stent	No	No	0.3 mo	Not reported
S10	Lopez 2023 Case 2	Endoscopic failure → surgery	Yes	Yes	ES, balloon, plastic stent	Yes	Yes (1.0 mo)	1.0 mo	Not reported
S12	Anebo 2026	Conservative	No	NA	—	No	NR	NR	Not reported
S13	Khan 2017	Conservative → surgery	No	NA	—	Yes	Yes (1.8 mo)	1.8 mo	Not reported
S14	Bellamlih 2021	Conservative	No	NA	—	No	No	4.0 mo	Not reported
S15	Bernshteyn 2020	Endoscopic	Yes	Yes	plastic stent	No	NR	NR	None reported
S16	Yanagaki 2020	Endoscopic → definitive surgery	Yes	Yes	—	Yes	No	5.0 mo	None reported
S17	Tabata 2020	Endoscopic	Yes	Yes	ES	No	No	24.0 mo	None reported
S18	Frauenfelder 2019 Case 1	Conservative	No	NA	—	No	No	NR	Not reported
S18	Frauenfelder 2019 Case 2	Conservative	No	NA	—	No	No	3.0 mo	Not reported
S19	Venkatanarasimha 2019	Conservative + percutaneous drainage	No	NA	—	No	No	48.0 mo	Not reported
S20	Oliveira 2019	Conservative	No	NA	—	No	No	4.0 mo	Not reported
S21	Miyajima 2018	Endoscopic	Yes	Yes	ES	No	No	0.4 mo	None reported
S22	Somani 2017	Endoscopic	Yes	Yes	ES	No	No	NR	None reported
S23	Rouet 2012	Endoscopic + percutaneous	Yes	Yes	ES	No	No	6.0 mo	None reported
S24	Kang 2014	Endoscopic	Yes	Yes	ES	No	Yes (6.0 mo)	12.0 mo	None reported
S25	Tobin 2018	Conservative	No	NA	—	No	NR	NR	Not reported
S26	Desai 2017	Conservative	No	NA	—	No	No	0.5 mo	Not reported

Abbreviations: ES, endoscopic sphincterotomy; NA, not applicable; NR, not reported.

### Efficacy

Technical success was achieved in 14/16 attempted procedures (87.5%, 95% CI 61.7–98.4; Table 4 ). Two failed at cannulation and were escalated to open surgery: one to choledochotomy with stent insertion and one to transduodenal sphincteroplasty and septoplasty. Durable endoscopic success was achieved in 12/16 (75.0%, 95% CI 47.6–92.7). Four ERCP recipients required surgical or percutaneous rescue (25.0%, 95% CI 7.3–52.4): the two cannulation failures, one patient who underwent three ERCP sessions before definitive laparoscopic biliodigestive bypass, and one who proceeded to extrahepatic bile duct resection with hepaticojejunostomy and diverticulectomy.

**Table 4:** Outcome estimates. Denominators differ by outcome and are stated explicitly. Confidence intervals are exact (Clopper–Pearson) except for the Kaplan–Meier estimates.

Outcome	Denominator population	*n* / *N*	Estimate	95% CI
ERCP technical success	ERCP recipients	14/16	**87.5%**	61.7–98.4
Durable endoscopic success	ERCP recipients	12/16	**75.0%**	47.6–92.7
Surgical or percutaneous rescue	ERCP recipients	4/16	**25.0%**	7.3–52.4
Post-ERCP pancreatitis	ERCP recipients, status reported	0/13	**0.0%**	0.0–24.7
Bleeding	ERCP recipients	1/16	**6.3%**	0.2–30.2
Perforation	ERCP recipients	0/16	**0.0%**	0.0–20.6
Any procedure-related complication	ERCP recipients, status reported	1/15	**6.7%**	0.2–31.9
30-day mortality	ERCP recipients	0/16	**0.0%**	0.0–20.6
Recurrence (crude proportion)	Cohort, status reported	3/21	**14.3%**	3.0–36.3
Recurrence at 6 months	Kaplan–Meier, whole cohort ( *n* = 20)	—	**26.7%**	8.9–64.3

Two classification decisions materially affect these figures and are declared here. In the patient who proceeded to hepatobiliary resection (Yanagaki 2020), the source report describes ERCP performed for diagnostic purposes before definitive surgery, and records no therapeutic endoscopic manoeuvre; we retained the patient as an ERCP recipient and as a durable-success failure, but if the procedure is judged diagnostic only, technical success becomes 13/15 (86.7%, 95% CI 59.5–98.3) and durable success 12/15 (80.0%, 95% CI 51.9–95.7). In Subramonian 2024, the concurrent laparoscopic cholecystectomy-treated coexisting cholelithiasis and was judged planned rather than endoscopic rescue. We do not report an overall clinical success proportion. Under the definition used in most previous reports—resolution of jaundice or cholangitis at any point and by any means—every patient in this cohort is a success by construction, including those cured surgically after endoscopic failure. Such a definition cannot separate the effect of the intervention under study from the effect of its rescue.

### Recurrence

Recurrence status was available for 21 patients. During the observation period, 3 of 21 patients (14.3%) experienced recurrence, while the remaining 18 patients (85.7%) remained recurrence-free until their last reported follow-up and were censored in the survival analysis.

Among the 20 patients with analysable follow-up, Kaplan–Meier analysis demonstrated an estimated recurrence-free survival of 93.3% at 1 month, 85.6% at 1.84 months, and 73.3% at 6 months ( Fig. 2 ). All documented recurrence events occurred within the first 6 months following treatment, after which the Kaplan–Meier curve remained stable throughout the remainder of the available follow-up period, with no additional recurrence events observed.

**Fig. 2:**
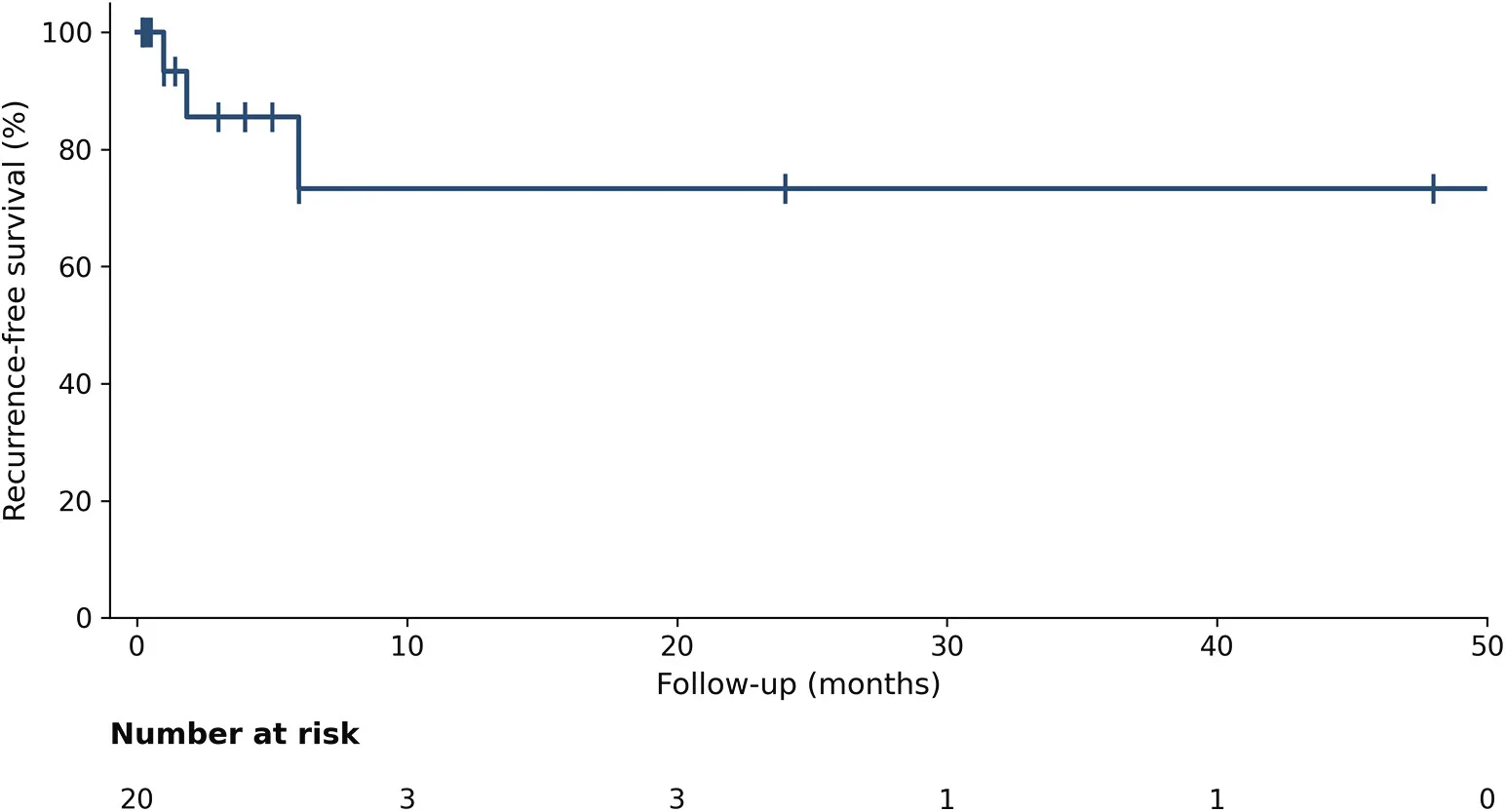
Kaplan–Meier estimate of recurrence-free survival among the 20 patients with analysable follow-up (whole analysis cohort). Vertical tick marks denote censored observations at the last reported contact. Three recurrences occurred, at 1.0, 1.84, and 6.0 months, giving estimated recurrence-free survival of 93.3% at 1 month, 85.6% at 1.84 months, and 73.3% at 6 months; no recurrence was observed thereafter. The number of patients at risk is shown beneath the plot. Median recurrence-free survival was not reached.

The number of patients at risk declined over time because of censoring, reflecting the variable duration of follow-up across the included reports. Consequently, recurrence-free survival estimates at later time points were based on progressively fewer patients under observation. The median recurrence-free survival was not reached, as recurrence-free survival remained above 50% throughout the follow-up period.

### Safety

One procedure-related adverse event occurred among 16 ERCP procedures: bleeding during needle-knife precut sphincterotomy that caused the index procedure to be abandoned, with a second session two days later achieving sphincterotomy and stent placement. Bleeding therefore occurred in 1/16 procedures (6.3%, 95% CI 0.2–30.2).

No post-ERCP pancreatitis, perforation or death was reported. These are zero-event findings in small denominators and must not be read as evidence of safety. With no events among 13 procedures in which pancreatitis status was reported, the data exclude only a true rate above 24.7%—that is, they remain compatible with a rate more than five times the approximately 4.6% reported in unselected ERCP populations. 10 The corresponding upper bounds are 20.6% for perforation and 20.6% for mortality. The safety of ERCP in Lemmel syndrome is undetermined by the present evidence.

### Completeness of Outcome Reporting

Outcome reporting was incomplete in a manner that constrains every estimate above. Across the 26-patient cohort, follow-up duration was reported for 20 patients (76.9%) and reached 3 months for 11 (42.3%); post-ERCP pancreatitis status was stated for 13 of 16 ERCP recipients; and recurrence status was stated for 21 of 26 patients. We report this pattern as a finding rather than solely as a limitation: the published evidence base for this condition does not currently support precise quantitative estimates of treatment effect.

### Risk of Bias

Of the 26 assessments corresponding to the analysis cohort, 25 were rated low risk of bias and one moderate (Somani 2017; displayed as unclear in Fig. 3 ); no report was rated high or critical ( Fig. 3 ). Item 7, identification and description of adverse events, was the weakest domain, satisfied in only 8 of 26 (31%)—most reports are silent on harms rather than reporting their absence. The single retrospective case series (Chen 2025), appraised with the ten-item JBI Critical Appraisal Checklist for Case Series, was rated moderate (unclear) risk, its weakest domains being consecutive and complete inclusion of participants and completeness of outcome reporting ( Fig. 4 ); as noted above, this source is excluded from the analysis cohort. In the pre-specified sensitivity analysis excluding the moderate-risk report, technical success was 13/15 (86.7%, 95% CI 59.5–98.3) compared with 14/16 (87.5%) in the full analysis; the conclusions were unchanged.

**Fig. 3:**
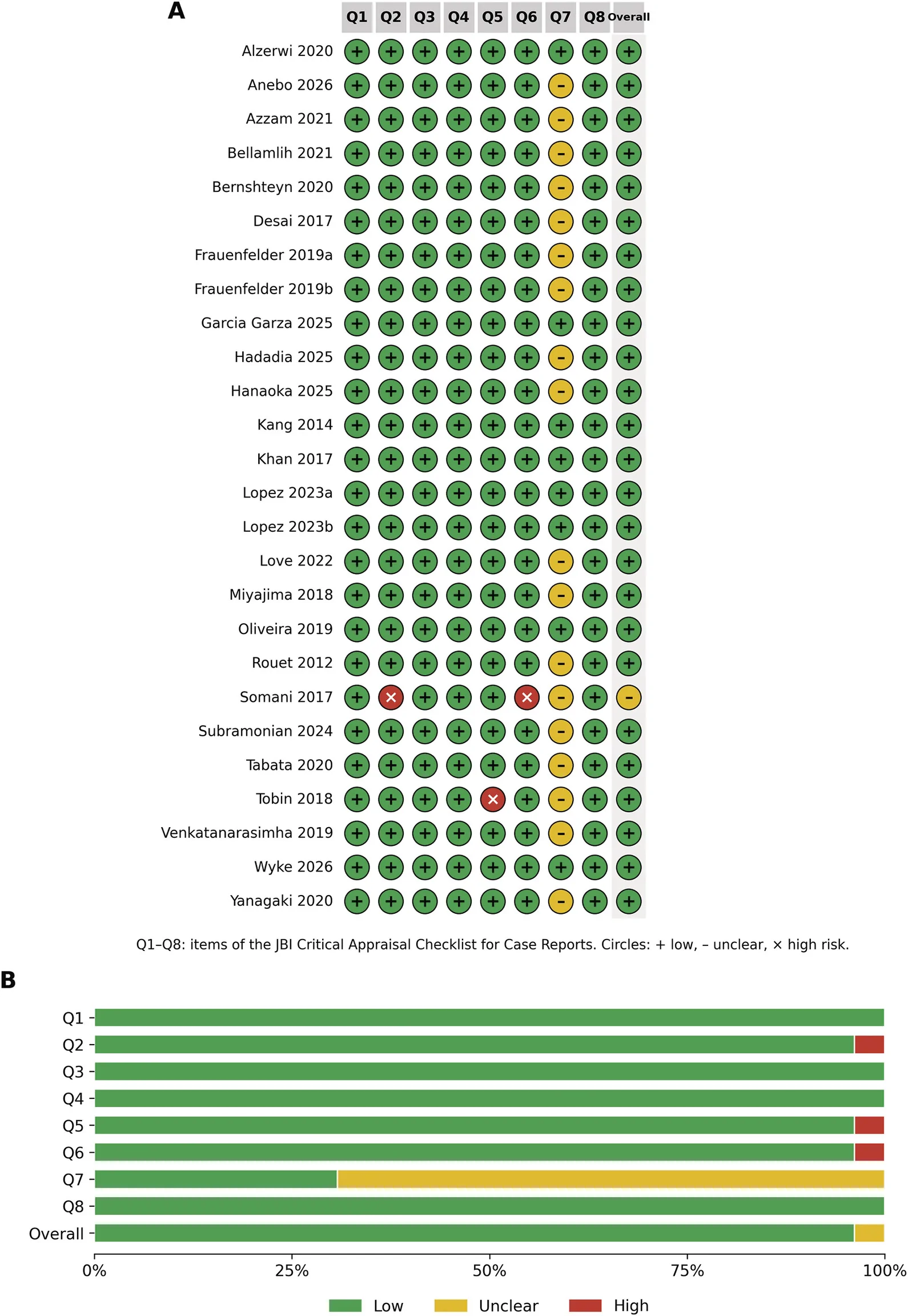
Methodological quality of the analysis-cohort case reports and individual cases (26 case-level appraisals from 24 publications; the two-patient series of Lopez and Frauenfelder are shown as individual cases), appraised with the Joanna Briggs Institute Critical Appraisal Checklist for Case Reports (eight items). ( **A** ) Per-item traffic-light plot (green, low risk; yellow, unclear; red, high risk). ( **B** ) Summary of item-level judgements across studies; item Q7 (identification and description of adverse events) is the weakest domain.

**Fig. 4:**
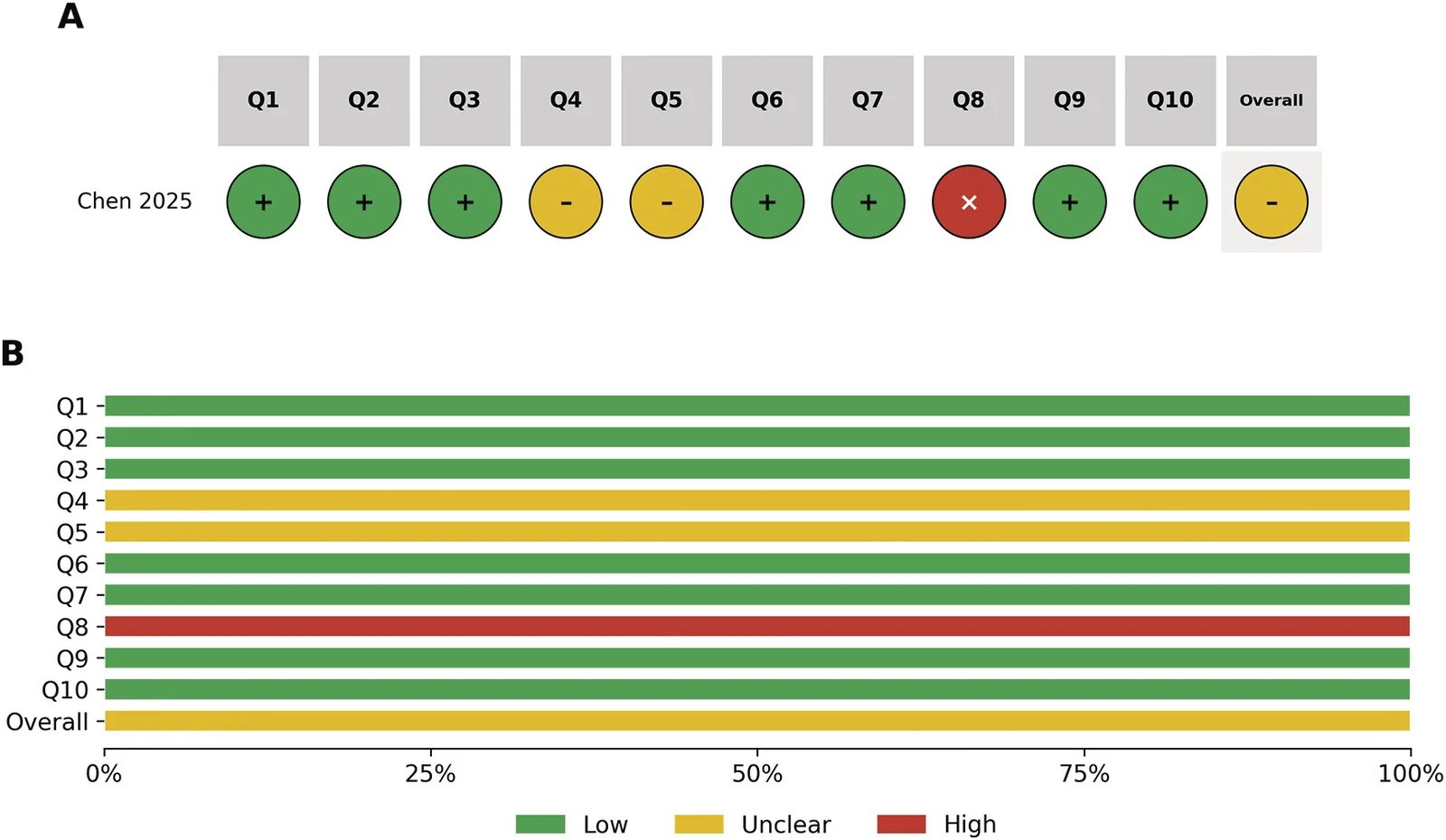
Methodological quality of the single retrospective case series (Chen 2025), appraised with the Joanna Briggs Institute Critical Appraisal Checklist for Case Series (ten items). ( **A** ) Per-item traffic-light plot and ( **B** ) summary of item-level judgements. This study is excluded from the analysis cohort.

### Certainty of Evidence

Certainty was rated very low for every outcome ( Table 5 ). Starting at low certainty as non-comparative observational evidence, all outcomes were downgraded further for risk of bias, imprecision, and publication bias. Although the JBI appraisal rated 25 of 26 case-level reports at low risk, that instrument reflects completeness of individual case reporting rather than the design-level risk of bias inherent to uncontrolled case reports, which cannot separate the treatment effect from selection and from rescue after endoscopic failure; certainty is therefore not raised on this basis. Imprecision was serious for every estimate, as denominators are small and confidence intervals very wide, and the zero-event outcomes—post-ERCP pancreatitis, perforation and 30-day mortality—have upper confidence limits that do not exclude clinically important harm. Publication bias is likely, since case reports are selectively published for favourable or unusual outcomes and no small-study test is computable for single-patient sources. Recurrence was additionally downgraded for the informative censoring described in the Methods. No outcome reached low or moderate certainty, and none of the estimates in Table 4 should be used as a point estimate for clinical decision-making.

**Table 5:** GRADE Summary of Findings: certainty of the evidence for each outcome. Effect estimates and 95% confidence intervals are reproduced from Table 4 . All outcomes derive from non-comparative observational data (case reports and small case series).

Outcome	Patients (n/N)	Effect estimate (95% CI)	Certainty (GRADE)	Reasons for downgrading
ERCP technical success	14/16	87.5% (61.7–98.4)	Very low ●○○○	a, b, c
Durable endoscopic success	12/16	75.0% (47.6–92.7)	Very low ●○○○	a, b, c
Surgical or percutaneous rescue	4/16	25.0% (7.3–52.4)	Very low ●○○○	a, b, c
Post-ERCP pancreatitis	0/13	0.0% (0.0–24.7)	Very low ●○○○	a, b, c
Bleeding	1/16	6.3% (0.2–30.2)	Very low ●○○○	a, b, c
Perforation	0/16	0.0% (0.0–20.6)	Very low ●○○○	a, b, c
Any procedure-related complication	1/15	6.7% (0.2–31.9)	Very low ●○○○	a, b, c
30-day mortality	0/16	0.0% (0.0–20.6)	Very low ●○○○	a, b, c
Recurrence (crude proportion)	3/21	14.3% (3.0–36.3)	Very low ●○○○	a, b, c
Recurrence at 6 months (Kaplan–Meier)	20 (KM)	26.7% (8.9–64.3)	Very low ●○○○	a, b, c, d

●○○○, GRADE certainty (very low). a, risk of bias: uncontrolled case reports and case series with high design-level risk of bias and frequently absent adverse-event reporting. b, imprecision: small denominators and wide confidence intervals; for zero-event outcomes the upper confidence limit does not exclude clinically important harm. c, publication bias: case reports are selectively published for favourable or unusual outcomes and no small-study test is computable. d, additionally downgraded for informative censoring in the Kaplan–Meier recurrence analysis.

## Discussion

This review identified 26 publications describing 46 patients, of whom 26 met the case definition with evaluable treatment data. Recurrence status was available for 21 patients, and Kaplan–Meier analysis was performed in the 20 patients with analysable follow-up. Crude recurrence occurred in 3 of 21 patients (14.3%), while Kaplan–Meier analysis estimated a cumulative recurrence of 26.7% by 6 months based on three recurrence events. All observed recurrence events occurred within the first 6 months of follow-up. These estimates should be interpreted cautiously because they are derived from a small number of patients with heterogeneous follow-up and are intended to describe the available follow-up rather than provide definitive estimates of long-term recurrence risk.

The overall pattern agrees with the earlier review by Love et al., which found that most reported patients improved with conservative or endoscopic treatment and that surgery was generally reserved for persistent or recurrent disease. 2 Chiang et al. similarly reported high cannulation success and a low complication rate in patients with cholangitis related to juxtapapillary diverticula, although a small proportion required surgery after failed endoscopic access. 44 More recent observational evidence is less uniformly favourable. In a 12-patient cohort, some elderly and comorbid patients had only partial improvement after stenting and one ultimately required surgery. 45 This difference may reflect the greater likelihood that routine cohorts include treatment failures and medically complex patients, whereas case reports preferentially describe successful or unusual outcomes.

Two points of overlap require explicit acknowledgement. First, the pooled review by Love et al. contains 46 cases, a number close to the 46 patients identified here; we counted only its index case, but a case-by-case cross-check against its case list remains outstanding and duplication cannot yet be formally excluded. Second, two Lemmel-specific reports discussed here—a 12-patient cohort with partial responses 45 and a case report of endoscopic management that was not feasible 46 —postdate our search and are not included. Both describe treatment failures, and their absence should be borne in mind when interpreting our findings, as treatment success may be overestimated and recurrence underestimated.

Evidence from the broader periampullary-diverticulum population supports the technical feasibility of ERCP but is only indirectly applicable. One meta-analysis found no significant increase in major ERCP complications among patients with periampullary diverticula, 47 whereas a more recent review reported modestly increased risks of post-ERCP pancreatitis and bleeding and identified intradiverticular papillae as a source of cannulation difficulty. 48 Differences in anatomy, operator expertise, study period, procedural technique and inclusion criteria may explain these contrasting findings. Patients with incidental periampullary diverticula should not be considered equivalent to those with true Lemmel syndrome.

Several mechanisms may explain the observed response. Lemmel syndrome may result from direct compression of the distal common bile duct, distortion of the ampulla, sphincter of Oddi dysfunction, or inflammation caused by impacted debris or an enterolith. Duodenobiliary reflux, bacterial colonisation, biliary stasis, and recurrent pigment-stone formation may also contribute. 2 5 Endoscopic stenting may maintain ductal drainage, sphincterotomy may reduce papillary resistance and facilitate clearance of sludge or stones, and lavage may relieve obstruction caused by impacted material. These remain biologically plausible hypotheses rather than proven explanations of treatment effect.

Clinically, ERCP appears reasonable for patients with significant obstruction, cholangitis, pancreatitis, persistent symptoms, or progressive cholestasis. Stable patients with mild symptoms may be considered for conservative management, whereas those with sepsis or ongoing obstruction are more likely to require biliary drainage. Complex papillary anatomy should prompt referral to an experienced pancreatobiliary endoscopist; ESGE guidance supports advanced cannulation strategies in difficult periampullary-diverticulum anatomy, although the supporting evidence is of low quality. 49 Standard ERCP precautions remain essential. 50 When endoscopic decompression fails, percutaneous, rendezvous or surgical approaches may be required. 46 Our findings add two points to this familiar advice. First, a proportion of patients required additional intervention after initial management. Second, Kaplan–Meier analysis of the whole analysable cohort estimated that approximately one in four patients experienced recurrence within 6 months, supporting planned clinical and biochemical follow-up rather than discharge after symptom resolution. Given the very low certainty of the evidence, these findings should be regarded as descriptive observations rather than precise estimates of recurrence risk.

This review was strengthened by a broad search strategy, inclusion of recent evidence, assessment of both efficacy and safety, and structured quality appraisal. Its conclusions remain limited by reliance on small, selected case reports. The evidence base is intrinsically weak and of low quality: it consists entirely of uncontrolled case reports and small case series, and all estimates should therefore be regarded as descriptive and hypothesis-generating rather than as a basis for firm clinical recommendations. Case reports are published because their outcomes are favourable or unusual, so these estimates describe the literature rather than the disease; no funnel plot or small-study test is meaningful with single-patient sources. Denominators differ by outcome because reporting is incomplete, and complete-case analysis assumes data are missing at random, which is unlikely for harms. Follow-up was short and inconsistent, so the Kaplan–Meier estimate was based on only three recurrence events and should be interpreted cautiously. The resulting confidence interval was wide, and the assumption of non-informative censoring may not be fully satisfied in a cohort assembled from case reports and case series. Treatment allocation was not randomised and was strongly confounded by indication, since milder presentations were managed conservatively; we therefore make no comparison between management strategies. Finally, two included patients had no symptoms recorded at presentation and two had a normal baseline bilirubin, which limits the interpretability of symptom- and biochemistry-based outcomes in those cases.

Future research should use prospective multicentre registries with standardised diagnostic criteria, treatment indications, outcome definitions, and long-term follow-up. Several questions remain unanswered and should be addressed prospectively: which mechanism—mechanical compression, sphincter of Oddi dysfunction, or papillary inflammation and fibrosis—produces symptoms in an individual patient, and why only a small minority of periampullary diverticula become symptomatic; how often the diverticulum makes papillary identification and cannulation difficult, and which cannulation techniques overcome this; the true rate of ERCP-related adverse events in this frequently elderly and septic population; and the incidence and timing of recurrence after endoscopic therapy. We propose a minimum reporting dataset for future Lemmel syndrome case reports: cannulation success; papilla position relative to the diverticulum using a named classification; sphincterotomy and stenting details; an explicit statement of each adverse event as present or absent; bilirubin trajectory with dates; and follow-up duration with the date of last contact. Adoption of such a dataset would allow a future synthesis to answer the questions this one cannot.

## Conclusion

In this patient-level synthesis of 26 patients with evaluable treatment data, ERCP-based therapy for symptomatic Lemmel syndrome was technically successful in 88% of attempts and achieved durable resolution without rescue in 75%, while one in four recipients required surgical or percutaneous rescue and Kaplan–Meier analysis estimated a cumulative recurrence of 26.7% at 6 months. Reported adverse events were few, but the denominators are too small to exclude clinically important harm, and certainty of evidence was very low for every outcome. ERCP remains a reasonable first-line approach in patients with significant obstruction, cholangitis or progressive cholestasis, provided that complex papillary anatomy prompts referral to an experienced pancreatobiliary endoscopist and that patients are counselled that recurrence may occur during the first 6 months of follow-up and that some patients may require a further procedure. The current evidence base does not support precise estimates of efficacy or safety, and prospective registry data are required.

## Data Availability

The dataset supporting the findings of this study is available from the corresponding author upon reasonable request.

## Declaration of GenAI Use

During preparation of the revised manuscript, the authors used Claude (Anthropic) to assist with language editing, and reorganisation of the figure and table legends. The authors reviewed and edited all output and take full responsibility for the content of the publication.
